# Herbivore dung quality affects plant community diversity

**DOI:** 10.1038/s41598-019-42249-z

**Published:** 2019-04-05

**Authors:** Elena Valdés-Correcher, Judith Sitters, Martin Wassen, Natacha Brion, Harry Olde Venterink

**Affiliations:** 1 Department of Biology, Vrije Universiteit Brussel, Pleinlaan 2, 1050 Brussels, Belgium; 2 Biogeco, INRA, University of Bordeaux, F-33610 Cestas, France; 3 Departments of Aquatic and Terrestrial Ecology, Netherlands Institute of Ecology (NIOO-KNAW), Droevendaalsesteeg 10, 6708 PB Wageningen, The Netherlands; 4 Environmental Sciences, Utrecht University, Heidelberglaan 2, 3584 CS Utrecht, The Netherlands; 5 Analytical, Environmental and Geochemistry, Vrije Universiteit Brussel, Pleinlaan 2, 1050 Brussels, Belgium

## Abstract

Nutrient availability is important for plant community composition and diversity, but most studies focus on inorganic nutrients. Far less is known about the impact of nutrients in organic forms such as herbivore dung. Here we show that dung of 11 European herbivore species varies widely in nitrogen (N) and phosphorus (P) concentrations, as well as in C:N:P ratios. We demonstrate that variation in dung quality of five herbivore species influences the diversity and composition of a mesocosm plant community. The impact of dung quality was at least as strong as, or stronger than, the effect of manipulating the quantity of dung by a factor six. Our study supports the hypothesis that both nutrient quantity and nutrient imbalances are important controlling factors for plant species diversity, and stresses the important role of herbivores on plant communities, not only via selective foraging, but also via stoichiometric variation of nutrients in their dung.

## Introduction

Nutrient availability is one of the main driving factors for composition and diversity of plant communities^1^. It also matters which nutrient – nitrogen (N), phosphorus (P) or potassium (K) – is growth-limiting for plant species composition and diversity, because some species compete most successfully for N, while others compete more successfully for P or K^2–4^. It was hypothesized that both the availability as well as the (im)balance of nutrients affect productivity-diversity relationships^5,6^. This hypothesis was tested across different ecosystems and approaches, whereby nutrient imbalance only weakly explained variation in species richness, and if so, only in freshwater and marine ecosystems, not in terrestrial habitats^7^. However, none of the terrestrial studies in this meta-analysis^7^ tested this hypothesis experimentally, and none considered nutrients in organic forms such as in herbivore dung.

**Figure 1:**
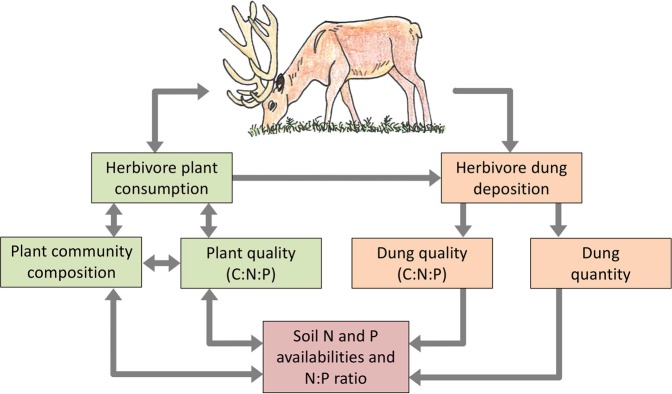
Conceptual framework on the stoichiometric impacts of herbivores on plants and soil through plant consumption and dung deposition. Dung quantity and quality (C:N:P) varies considerably among herbivore species, influencing release rates of N and P to the soil and hence soil N and P availabilities and N:P ratio, in turn impacting competitive interactions between plants and plant quality. Herbivores simultaneously influence plant community composition through selective feeding on the most nutritious plants, which might change soil N and P availabilities through changes in litter quality.

Mammalian herbivores provide an important source of nutrients to plants through dung and urine deposition^8,9^. Variation in the spatial distribution of the quantity of dung has been observed to affect plant community productivity and composition^10–12^. It is also increasingly recognized that dung quality, in terms of the C:N:P stoichiometry, might vary considerably between herbivore species^13^. This variation partly corresponds to differences in the herbivores’ feeding strategy (e.g., browsers *vs*. grazers), and thus the quality of plants they consume (Fig. 1), their digestive physiology (e.g., ruminant *vs*. non-ruminants), and body weight^8,13,14^. Dung quality also varies among seasons^15,16^ reflecting changes in abundance and quality of the vegetation^17^. The variation in dung C:N:P stoichiometry in turn has a large impact on the return rates of N and P to the soil^13^, likely influencing N and P availabilities in the soil and competitive interactions between plants, and thus impacting the diversity and composition of plant communities^18^ (Fig. 1). However, so far only a few studies have compared dung C:N:P stoichiometry among mammalian herbivore species, and none to our knowledge have examined the impact of this variation in dung quality on plant species competition.

Here, we determine the variation in dung quality (C:N:P) among a group of common mammalian herbivore species in Europe. Thereto, we collected fresh dung of 11 herbivore species in three nature reserves in The Netherlands and Belgium, both in winter and in spring, and we measured total C, N and P concentrations in it. We also test whether the observed variation in dung quality was large enough to have substantial ecological consequences – for instance to influence plant community composition. We carried out a mesocosm experiment with species mixtures of six European grassland plants: two leguminous forbs *Trifolium pratense* and *Lotus corniculatus*, two grasses *Holcus lanatus* and *Agrostis capillaris*, and two non-leguminous forbs *Taraxacum officinale* and *Achillea millefolium*. We fertilized the mesocoms with dung (collected in winter) of one of the following five herbivores: European bison, cow, horse, fallow deer or rabbit, in either a low or a high quantity (5 or 30 gram dry dung per mesocosm). Hence, we varied both the quantity and imbalance of the nutrients provided to the plants in line with the concept of Cardinale and others^5^. We expected to find variation in dung quality in the field study, but had no clue whether this would be large enough to affect diversity of the plant community in a short-term mesocosm experiment. We did expect that dung quantity would stimulate community productivity, in line with the practice of organic farming, and that a higher productivity might lead to a reduced diversity through reduced light availability^1^.

## Results and Discussion

### Variation in dung quality

Dung of the 11 mammalian herbivore species varied considerably (factor two to three) in N and P concentrations, in N:P ratio, and in ratios with C (C:N, C:P) (Supplementary Tables 1 and 2). The dung quality also varied among seasons, likely due to differences in food quality. Generally, N and P concentrations were higher in spring than in winter, whereas C:N and C:P were lower. Variation in dung quality between herbivore species was also related to their feeding strategy (Suppl. Table 1) and body weight (Suppl. Fig. 1). Hence, this field study showed that dung quality of European herbivores varies considerably among species, seasons and environmental conditions, due to variation in diet, size and/or digestive physiology. This result is fully consistent with observations from African savanna^13,19,20^, which is probably the most intensively studied ecosystem on Earth in this respect.

### Effect of dung quality and quantity on plant diversity and productivity

**Figure 2:**
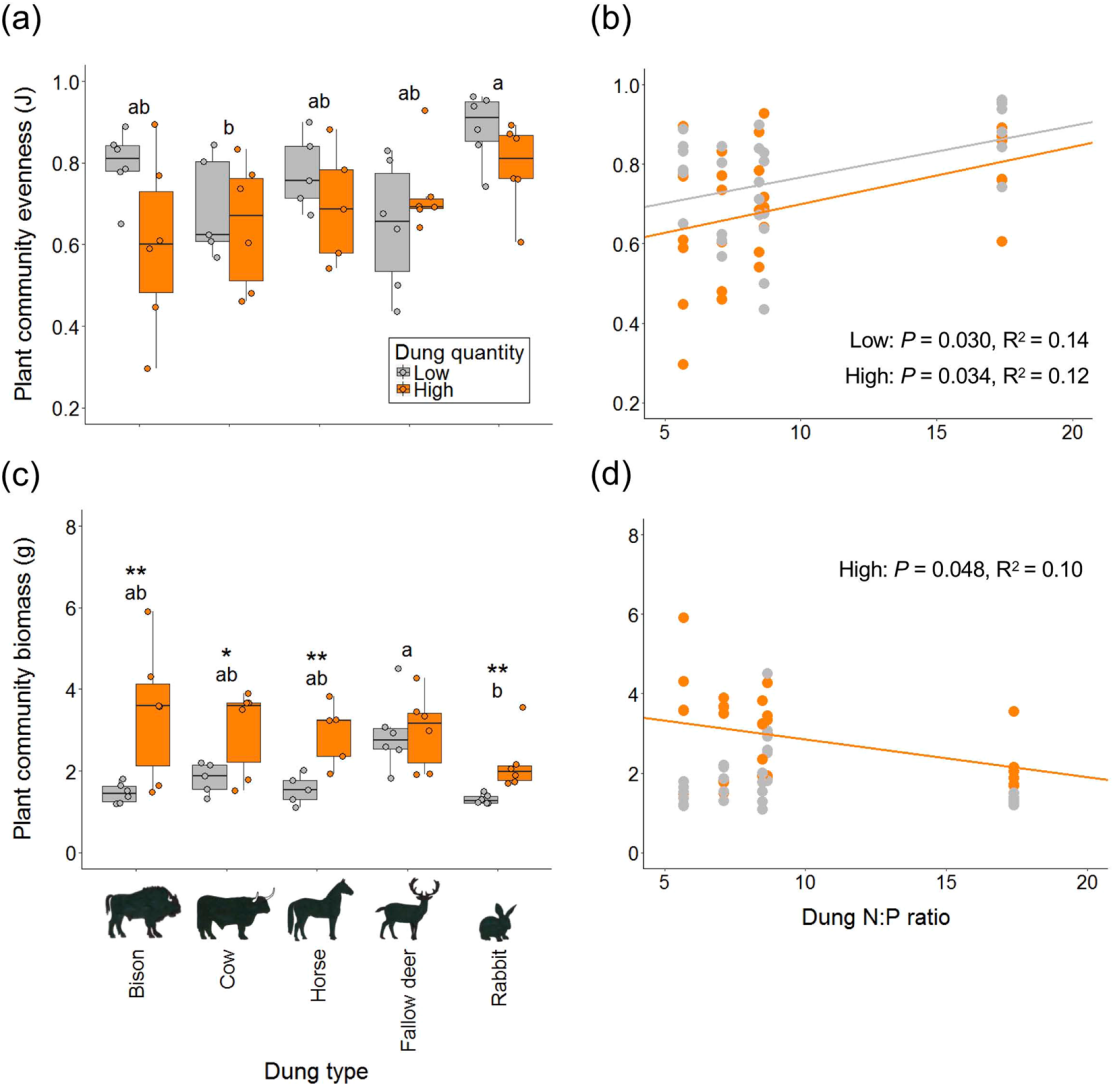
The effect of dung quality and dung quantity on the evenness and total biomass of an experimental plant community. Evenness (**a**) and total biomass of plant communities (**c**) in mesocosms fertilized with a low quantity (grey boxplots and points, 5 g dry dung per mesocosm) or a high quantity (orange boxplots and points, 30 g dry dung per mesocosm) of dung from different European herbivores (European bison, cow, horse, fallow deer and rabbit). Bars show means ± SE of 6 replicates, except for low-cow, low-horse and high-horse (n = 5). Values not sharing the same letter indicate significant differences between dung types combining low and high quantity bars, since there was no significant dung type x dung quantity interaction (results from ANOVA; Table 2). Asterisks indicate significant differences in total plant biomass between dung quantities (with ^*^P < 0.05, ^**^P < 0.01). Plant community evenness (**b**) and community biomass (**d**) plotted against the N:P ratio of the supplied dung. Grey points show low dung quantity and orange points high dung quantity. Significant linear regression lines are shown. All plant communities consisted of the same six species with two leguminous forbs (*Trifolium pratense* and *Lotus corniculatus*), two grasses (*Holcus lanatus* and *Agrostis capillaris*), and two other forbs (*Taraxacum officinale* and *Achillea millefolia*).

The dung N:P ratio of the five species used in the mesocosm experiment varied threefold, from 5.7 for European bison to 17.4 for rabbit (Table 1). This variation was clearly large enough to influence plant community composition. After two months, dung quality (herbivore species, dung type, N:P ratio) had significantly affected the relative abundances of the six plant species (significant interaction with plant species in Suppl. Table 3, Suppl. Figs 2 and 3), as well as plant community evenness and biomass (Table 2, Fig. 2). Rabbit dung with N:P ratio of 17 maintained the most diverse plant community (evenness 0.8–0.9), probably because it provided the most balanced N:P ratio relative to the stoichiometric demand of plants. The critical N:P ratios whereby plant growth is equally limited by N and P most commonly lie between 10 and 20^21^. Dung with lower N:P ratios stimulated communities with higher abundances of the leguminous forbs, especially *T*. *pratense*, while the grasses (*A*. *capillaris* and *H*. *lanatus*) grew best under dung with higher N:P ratios (Suppl. Figs. 2 and 3). The high dominance of *T. pratense* compared to the non-legumes under low N:P supply ratio, hence under N-limited conditions^21^, was likely related to its ability to symbiotically fix atmospheric N and explore this alternative source of N. Its roots indeed contained many nodules, many more than the roots of the other legume species *L*. *corniculatus*, and the number of nodules was positively correlated to relative abundance of the legume species (Suppl. Fig. 4). Nodule numbers may illustrate that *L*. *corniculatus* was able to fix less N than *T*. *pratense*, either because it was outcompeted by *T*. *pratense* or because other factors limited its N fixation. With higher N:P dung, particularly of rabbits, *T*. *pratense* was less able to outcompete other species, such as the grass *H*. *lanatus* (Suppl. Figs 2 and 3).

**Table 1:** Variation in dung quality among several common European herbivore species.

Herbivore species	n	C (mg/g)	N (mg/g)	P (mg/g)	C:N ratio	C:P ratio	N:P ratio
European bison	6	445 ± 13^a^	11.3 ± 0.5^c^	2.13 ± 0.26^a,b^	39.8 ± 1.0^a^	224 ± 25^b,c^	5.67 ± 0.67^b^
Cow	7	480 ± 4^a^	10.6 ± 0.3^c^	1.67 ± 0.26^a,c^	45.7 ± 0.9^a^	317 ± 33^a,b^	7.00 ± 0.76^b^
Horse	5	473 ± 6^a^	10.7 ± 0.4^c^	1.29 ± 0.11^b,c^	44.2 ± 1.5^a^	376 ± 30^a,b^	8.46 ± 0.54^b^
Fallow deer	12	432 ± 10^b^	19.7 ± 0.8^a^	2.76 ± 0.39^a^	22.5 ± 1.4^c^	187 ± 21^c^	8.65 ± 1.16^b^
Rabbit	10	438 ± 11^b^	16.6 ± 0.4^b^	1.04 ± 0.10^c^	26.4 ± 0.6^b^	462 ± 51^a^	17.4 ± 1.82^a^

Dung C, N and P concentrations and C:N, C:P and N:P ratios (mean ± SE; n = 5–12) for several common European herbivore species from Kennemerduinen in winter. This dung was used in the mesocosm experiment. Values with different letters indicate significant differences between herbivore species (Tukey test, *P* < 0.05).

**Table 2:** Differences in plant community evenness and biomass under different dung types and quantities.

Source of variation	Dung type (DT)		Dung quantity (DQ)		DT x DQ	
	*F*-ratio	*P*-value	*F*-ratio	*P*-value	*F*-ratio	*P*-value
Community evenness	**3**.**1**	**0**.**026**	3.6	0.064	1.7	0.174
Community biomass	**4**.**4**	**0**.**004**	**30**.**4**	**<0**.**001**	2.3	0.076

Two-way ANOVA results for the effects of dung type (herbivore species) and dung quantity on the evenness index and total biomass of the experimental plant community.

As expected, dung quantity affected plant community composition through a strong effect on productivity (albeit not for fallow deer dung, for which we have no better explanation than biological variation or experimental bias), and a smaller effect on evenness (the latter *P* = 0.064; Table 2, Fig. 2). The negative effect of community biomass on diversity (Suppl. Fig. 5) was likely due to reduced light conditions^1^. Noteworthy, community biomass increased with decreasing dung N:P ratio, but only at high dung quantity (Fig. 2d). Under high supply of low N:P dung, *T*. *pratense* was most abundant (Suppl. Fig. 2) and could likely fix the most atmospheric N (Suppl. Fig. 4), enabling the highest community productivity. While biomass only decreased with dung N:P at high quantity, community evenness increased with dung N:P at both high and low quantity (Fig. 2b,d). This is an important result, since it illustrates that the positive effect of dung quality (N:P) on evenness was not just an indirect effect of increased light through reduced biomass, but also a direct effect, at least under low dung supply.

The results of this study support the hypothesis^5^ that both nutrient quantity and imbalance influence the diversity and productivity of a plant community (Table 2, Fig. 2.), whereby biomass response depends on plant functional type; non-leguminous forbs responsed more strongly to nutrient quantity, while leguminous forbs and grasses responsed more strongly to nutrient imbalance (Suppl. Fig. 2). The outcome of our study is novel for three reasons. First, it is the first support for this hypothesis from a terrestrial community; so far it was only supported for freshwater and marine ecosystems^5,7^. Second, it is supported by means of an experimental approach, whereas previous support was derived from field monitoring^5,7^. Third, and most importantly, for the first time we showed that variation in quantity and quality of natural organic fertilisers (herbivore dung), affected plant species performance in a community experiment, whereas so far this was only examined for mineral (inorganic) forms of nutrients. Furthermore, the effect of the natural variation in dung quality (a factor three in N:P ratio) on plant community diversity was as least as strong as, or stronger than, the effect of manipulating the amount of dung by a factor six (Fig. 2b). This clearly shows that not only the spatial deposition pattern (quantity) of herbivore dung is important but also the highly variable quality (C:N:P stoichiometry) among herbivore dung. This stoichiometric variation should be considered when assessing the role of herbivores on ecosystem processes and plant communities.

It is likely that the influence of dung quantity and quality on plant communities will be far more difficult to detect under natural circumstances than in our experiment, because herbivores also affect plant community composition through consumption^22^ (cf. Fig. 1). Herbivores can influence plant community composition through selective feeding on the most nutritious plants, often legumes with high foliar N and P concentrations. Selective consumption of these palatable plant species can provide a competitive advantage for unpalatable plant species of low quality, which in turn produce low quality litter that is slowly decomposed^23,24^. Through this pathway herbivores may decrease soil N and P availabilities and hence also impact plant processes (Fig. 1). Therefore, field studies analyzing the effects of herbivores on plant communities should focus on uncoupling the effects of selective plant consumption and dung deposition by herbivores as both have a potential impact on the spatial redistribution of nutrients and their balances, and hence on plant community processes. Such field studies are needed to determine the relative importance of dung C:N:P stoichiometry for plant community composition in comparison to other herbivore effects, such as selective consumption, trampling, avoidance of dung and urine patches, which could obviously not be included in our short-term experiment with only one artificial plant community.

## Methods

### Dung collection and nutrient analyses

5–12 samples of freshly deposited dung (a max. of three days old) of fallow deer, roe deer, red deer, two different breeds of horse (Konik horse, Shetland pony), three different breeds of cow (Highland, Angus, Heck), European bison, rabbit, hare, wild boar, sheep and goose were collected during winter (on 19, 20 and 22 January 2015) and spring (on 14, 15 and 19 April 2015) in three nature reserves (the Kennemerduinen located in the Netherlands, The Zwarte Beek Vallei Natural Reserve located in Belgium and the Nature Reserve Oostvaardersplassen located in the Netherlands). Experienced rangers helped with the collection and identification of dung. After the collection, fresh dung was dried at 70 °C for 48 hours and subsequently ground in the lab at Vrije Universiteit Brussel in Belgium with a Retsch Mixer Mill MM300 (Westburg). Total C and N contents in dung were analyzed with an elemental analyser (Thermo EA Flash 1112). Total P was analyzed using a modification of the combustion and hot HCl extraction procedure of Andersen^25^ according to Johengen^26^.

### Mesocosm experiment

On 21–22 February 2015, a total of six seedlings (six species mixture) of equal size were selected and planted in 3.5 L mesocoms filled with quartz sand containing no detectable N or P. Each mesocosm was placed on a separate dish, which were watered daily with deionised water. Seedling biomass at the start of the experiment was: 11 ± 0.66 mg for *Trifolium pratense*, 13.1 ± 1.41 mg for *Taraxacum officinale*, 4.7 ± 0.48 mg for *Holcus lanatus*, 1.2 ± 0.24 mg for *Agrostis capillaris*, 6.6 ± 1.22 mg for *Lotus corniculatus* and 10.6 ± 1.30 mg for *Achillea millefolium* (means ± SE, n = 5). The distribution of plants in each mescocosm followed the same pattern with equal distances between each plant. A mesocosm received 5 or 30 gram dried and ground dung from one of the following species: European bison, cow, horse, fallow deer or rabbit (see Table 1 for quality). Dung added to the mesocosms was mixed with 8 g of grassland soil in the upper layer of each mesocosm in order to provide soil microbes for dung decomposition and plant-microbe interactions (Rhizobia and mycorrhiza). The total number of mesocosms was: dung of 5 herbivore species $\times$ 2 dung quantity levels $\times$ 6 replicates = 60 mesocosms. From 25 February 2015 onwards until the end of the experiment, essential nutrients (but not N and P) were given as salt solutions in constant non-limiting amounts to all the mesocosms once a week. The proportions of essential nutrients given to the plants were according to Güsewell and Bollens^27^. The 60 mesocosms were randomly placed in the greenhouse at Vrije Universiteit Brussel in blocks with every treatment occurring once within each block, with an average temperature of 26 °C, 40% relative humidity and additional light period from 7 am until 11 pm. During the experiment, dead leaves and shoots were collected. After two months, the plants were harvested (both roots and shoots) and dried at 70 °C for 48 hours and weighed. We skipped three mesocosms (1 ‘low-cow’, 1 ‘low-horse’ and 1 ‘high-horse’) from the dataset because one or more plants had died during an early stage of the experiment.

### Statistical analyses

We analysed the effects of herbivore species, season and their interaction on dung quality (C, N, P concentrations and ratios) with two-way unbalanced ANOVAs due to differences in number of dung samples per herbivore species and/or season. We performed a box-cox transformation on the dung quality data and we obtained type II Sums of Squares using the function ‘Anova’ in the ‘car’ package in R^29^ (version 3.4.2). For the dung samples of the five herbivore species used in the mesocosm experiment these ANOVAs were followed by Tukey-Kramer HSD tests. The evenness of the experimental plant community was calculated based on Pielou’s evenness index^28^. To test for the effects of herbivore dung type and dung quantity on the evenness and total biomass of the experimental plant community we used two-way unbalanced ANOVAs due to the missing mesocosms (again type II SS with function ‘Anova’). Community biomass was log-transformed to meet assumptions of normality and homogeneity. To examine the effects of the dung treatments on the relative abundance of plants per mesocosm we performed a redundancy analysis (RDA) using the ‘vegan’ package^30^. We used herbivore dung N:P and dung quantity (recoded as 5 for low and 30 for high) as numeric explanatory variables without interaction. The significance of effects was tested using Monte Carlo permutation tests (999 permutations). Additionally, we analysed the effects of plant species, dung type, dung quantity and their interactions on the relative abundance of plants per mesocosm with a linear-mixed effects model. We used mesocosm identification number as random factor to account for the non-independence of species grown in the same mesocosm^3^. Relative abundances were square root and arcsine-transformed to meet assumptions of normality and homogeneity. We performed an ANOVA on this model, which was followed by Tukey-Kramer HSD tests to examine the effects of dung type and quantity on the relative abundance of the different plant species (due to significant interactions between plant species x dung type and plant species x dung quantity). Linear regression models were used to analyze the relationship between the evenness index and dung N:P ratio, and between total biomass and dung N:P ratio.

## Supplementary information

Supplementary tables and figures

## Data Availability

The datasets collected and analyzed for this study are available in figshare at https://figshare.com/s/99148faf2526be768f62 and https://figshare.com/s/87c6e2fff1b6a480db53.
