# Supplementary material for: Herbivore dung quality affects plant community diversity
Source: Sci Rep. 2019 Apr 5;9:5675. doi: 10.1038/s41598-019-42249-z (PMC6450897; doi:10.1038/s41598-019-42249-z)
Supplement: Supplementary file 1 — Supplementary tables and figures [file 41598_2019_42249_MOESM1_ESM.pdf]

## Supplementary information for

# Herbivore dung quality affects plant community diversity

Elena Valdés-Correcher[1,2]*, Judith Sitters[1,3]*, Martin Wassen[4], Natacha Brion[5] and Harry Olde Venterink[1]*#

[1] Department of Biology, Vrije Universiteit Brussel, Pleinlaan 2, 1050 Brussels, BELGIUM
[2] Biogeco, INRA, University of Bordeaux, F-33610 Cestas, FRANCE
[3] Departments of Aquatic and Terrestrial Ecology, Netherlands Institute of Ecology (NIOO-KNAW), Droevendaalsesteeg 10, 6708 PB Wageningen, THE NETHERLANDS
[4] Environmental Sciences, Utrecht University, Heidelberglaan 2, 3584 CS Utrecht, THE NETHERLANDS
[5] Analytical, Environmental and Geochemistry, Vrije Universiteit Brussel, Pleinlaan 2, 1050 Brussels, BELGIUM

*These authors contributed equally to this work
[#]Corresponding author (e-mail: harry.olde.venterink@vub.be)

**This pdf file includes:**

Supplementary Table 1. Variation in dung quality among herbivore species, seasons and nature reserves.
Supplementary Table 2. Differences in dung quality between herbivore species and season per nature reserve.
Supplementary Table 3. Relative abundance of plants affected by plant species, dung type and dung quantity.
Supplementary Fig. 1. Dung quality in relation to herbivore body weight.
Supplementary Fig. 2. Ordination biplot showing the result of RDA analysis of the relative abundances of plants in mesocosms under different dung treatments.
Supplementary Fig. 3. Effect of dung type and quantity on the relative abundance of the different plant species in an experimental plant community.
Supplementary Fig. 4. Legume abundance in relation to nodulation.
Supplementary Fig. 5. Plant community evenness plotted against plant community biomass.

**Supplementary Table 1. Variation in dung quality among herbivore species, seasons and nature reserves.** Dung C, N and P concentrations and C:N, C:P and N:P ratios (mean ± SE; n = 5-12) for several common European herbivore species from Kennemerduinen, Zwarte Beek Vallei and Oostvaardersplassen in winter and spring. DP = Digestive Physiology with R = Ruminant or N = Non-ruminant; FS = Feeding Strategy with G = Grazer, M = Mixed feeder or O = Omnivore.

| Herbivore species | n (winter/ spring) | DP and FS | C (mg/g) | | N (mg/g) | | P (mg/g) | | C:N ratio | | C:P ratio | | N:P ratio | |
|---|---|---|---|---|---|---|---|---|---|---|---|---|---|---|
| | | | Winter | Spring | Winter | Spring | Winter | Spring | Winter | Spring | Winter | Spring | Winter | Spring |
| ***Kennemerduinen (NL)*** | | | | | | | | | | | | | | |
| European bison | 6 / 5 | R-M | 445 ± 13 | 414 ± 29 | 11.3 ± 0.5 | 20.3 ± 2.6 | 2.13 ± 0.26 | 3.48 ± 0.27 | 39.8 ± 1.0 | 21.4 ± 2.4 | 224 ± 25 | 121 ± 9 | 5.67 ± 0.67 | 5.97 ± 0.85 |
| Cow (Highland cattle) | 7 / 5 | R-G | 480 ± 4 | 438 ± 5 | 10.6 ± 0.3 | 22.7 ± 1.2 | 1.67 ± 0.26 | 2.66 ± 0.49 | 45.7 ± 0.9 | 19.5 ± 1.0 | 317 ± 33 | 186 ± 301 | 7.10 ± 0.76 | 9.95 ± 2.08 |
| Horse (Konik horse) | 5 / 9 | N-G | 473 ± 6 | 454 ± 10 | 10.7 ± 0.4 | 17.5 ± 0.7 | 1.29 ± 0.11 | 3.02 ± 0.09 | 44.2 ± 1.5 | 26.1 ± 0.8 | 376 ± 30 | 151 ± 6 | 8.46 ± 0.54 | 5.81 ± 0.24 |
| Horse (Shetland pony) | 7 / 5 | N-G | 440 ± 50 | 484 ± 6 | 8.39 ± 1.2 | 16.0 ± 0.8 | 1.34 ± 0.08 | 4.98 ± 0.39 | 54.1 ± 2.9 | 30.4 ± 1.2 | 344 ± 47 | 100 ± 7 | 6.51 ± 0.94 | 3.28 ± 0.23 |
| Fallow deer | 12 / 6 | R-M | 432 ± 10 | 457 ± 11 | 19.7 ± 0.8 | 35.1 ± 1.8 | 2.76 ± 0.39 | 2.74 ± 0.26 | 22.5 ± 1.4 | 13.1 ± 0.5 | 187 ± 21 | 172 ± 13 | 8.65 ± 1.16 | 13.1 ± 0.84 |
| Rabbit | 10 / 5 | N-M | 438 ± 11 | 415 ± 23 | 16.6 ± 0.4 | 22.8 ± 1.3 | 1.04 ± 0.10 | 2.54 ± 0.46 | 26.4 ± 0.6 | 18.6 ± 1.6 | 462 ± 51 | 184 ± 33 | 17.4 ± 1.82 | 9.93 ± 1.41 |
| Goose | 5 | N-G | | 378 ± 35 | | 17.2 ± 2.1 | | 2.12 ± 0.30 | | 22.4 ± 1.4 | | 194 ± 36 | | 9.02 ± 2.21 |
| ***Zwarte Beek Vallei (BE)*** | | | | | | | | | | | | | | |
| Cow (Angus cattle) | 6 | R-M | | 457 ± 13 | | 26.0 ± 0.8 | | 8.66 ± 0.8 | | 17.6 ± 0.4 | | 55 ± 5 | | 3.11 ± 0.25 |
| Horse (Konik horse) | 5 / 5 | N-M | 443 ± 5 | 476 ± 14 | 10.5 ± 0.3 | 23.1 ± 1.0 | 2.69 ± 0.21 | 5.81 ± 0.73 | 42.4 ± 1.3 | 20.8 ± 1.2 | 169 ± 14 | 87 ± 11 | 3.96 ± 0.21 | 4.21 ± 0.52 |
| Sheep | 6 / 5 | N-G | 505 ± 7 | 464 ± 14 | 18.4 ± 0.3 | 39.9 ± 3.5 | 2.35 ± 0.36 | 3.16 ± 0.20 | 27.4 ± 0.7 | 12.0 ± 1.1 | 242 ± 37 | 150 ± 11 | 8.90 ± 1.48 | 12.8 ± 1.4 |
| Wild boar | 5 / 5 | N-O | 403 ± 18 | 521 ± 6 | 25.1 ± 1.5 | 22.3 ± 0.4 | 9.80 ± 2.0 | 7.81 ± 1.8 | 16.2 ± 0.8 | 23.4 ± 0.5 | 51 ± 14 | 89 ± 29 | 3.08 ± 0.70 | 3.79 ± 1.17 |
| Roe deer | 10 / 12 | R-M | 508 ± 3 | 490 ± 9 | 23.6 ± 0.5 | 42.8 ± 1.3 | 3.02 ± 0.38 | 8.76 ± 0.61 | 21.6 ± 0.5 | 11.5 ± 0.3 | 192 ± 22 | 59 ± 5 | 8.96 ± 1.09 | 5.20 ± 0.45 |
| Hare | 5 | N-M | 492 ± 5 | | 22.4 ± 2.1 | | 3.28 ± 1.16 | | 22.8 ± 2.3 | | 247 ± 70 | | 10.0 ± 2.29 | |
| ***Oostvaardersplassen (NL)*** | | | | | | | | | | | | | | |
| Cow (Heck cattle) | 5 / 5 | R-G | 360 ± 14 | 252 ± 6 | 12.8 ± 0.7 | 17.3 ± 1.2 | 1.79 ± 0.50 | 3.70 ± 0.81 | 28.8 ± 2.8 | 14.8 ± 0.8 | 379 ± 198 | 101 ± 41 | 13.1 ± 6.50 | 6.97 ± 2.93 |
| Horse (Konik horse) | 5 / 5 | N-G | 316 ± 16 | 241 ± 8 | 12.4 ± 0.4 | 16.8 ± 1.0 | 2.74 ± 0.11 | 4.27 ± 0.34 | 25.4 ± 1.2 | 14.4 ± 0.4 | 117 ± 10 | 57.5 ± 3.4 | 4.57 ± 0.22 | 3.97 ± 0.17 |
| Red deer | 10 / 5 | R-M | 315 ± 27 | 351 ± 15 | 16.2 ± 0.9 | 25.8 ± 0.7 | 3.20 ± 0.31 | 5.29 ± 1.27 | 19.3 ± 1.0 | 13.6 ± 0.5 | 113 ± 18 | 122 ± 66 | 5.63 ± 0.67 | 8.97 ± 4.84 |

**Supplementary Table 2. Differences in dung quality between herbivore species and season per nature reserve.**

Two-way ANOVA results (*F*-ratios and significance levels) for the effects of herbivore species and season on dung nutrient concentrations (mg/g) and ratios in the natural reserves Kennemerduinen, Zwarte Beek Vallei, and Oostvaardersplassen.

| Source of variation | d.f. | C | N | P | C:N | C:P | N:P |
|---|---|---|---|---|---|---|---|
| *Kennemerduinen* | | | | | | | |
| Herbivore species (HS) | 6 | 4.2** | 40.8*** | 10.2*** | 74.3*** | 6.6*** | 12.9*** |
| Season (S) | 1 | n.s. | 207.0*** | 92.8*** | 359.3*** | 69.7*** | n.s. |
| HS x S | 4 | n.s. | 3.6** | 6.7*** | 6.1*** | 4.6** | 5.5*** |
| *Zwarte Beek Vallei* | | | | | | | |
| Herbivore species (HS) | 5 | 6.6*** | 80.4*** | 9.3*** | 51.6*** | 10.0*** | 15.4*** |
| Season (S) | 1 | n.s. | 323.6*** | 31.0*** | 263.4*** | 26.2*** | n.s. |
| HS x S | 3 | 22.6*** | 49.5*** | 7.6*** | 66.5*** | 10.3*** | 3.9* |
| *Oostvaardersplassen* | | | | | | | |
| Herbivore species (HS) | 2 | n.s. | 32.7*** | n.s. | 10.6*** | n.s. | n.s. |
| Season (S) | 1 | 4.9* | 82.0*** | 11.9** | 124.6*** | 10.4** | n.s. |
| HS x S | 2 | 6.1** | 9.5*** | n.s. | n.s. | n.s. | n.s. |

*Significance levels: *P < 0.05, ** P < 0.01, *** P < 0.001, n.s. P > 0.05; d.f.: degrees of freedom*

**Supplementary Table 3. Relative abundance of plants affected by plant species, dung type and dung quantity.**

Three-way ANOVA results (*F*-ratios and significance levels) for the effects of plant species, dung type (herbivore species) and dung quantity on the relative abundance of plants in the experimental plant community.

| | | Relative abundance | |
|---|---|---|---|
| Source of variation | d.f. | *F*-ratio | *P*-value |
| Plant species (PS) | 5 | **211.8** | **<0.001** |
| Dung type (DT) | 4 | 0.8 | 0.536 |
| Dung quantity (DQ) | 1 | 1.0 | 0.318 |
| PS x DT | 20 | **3.1** | **<0.001** |
| PS x DQ | 5 | **8.0** | **<0.001** |
| DT x DQ | 4 | 0.5 | 0.750 |
| PS x DT x DQ | 20 | 0.8 | 0.676 |

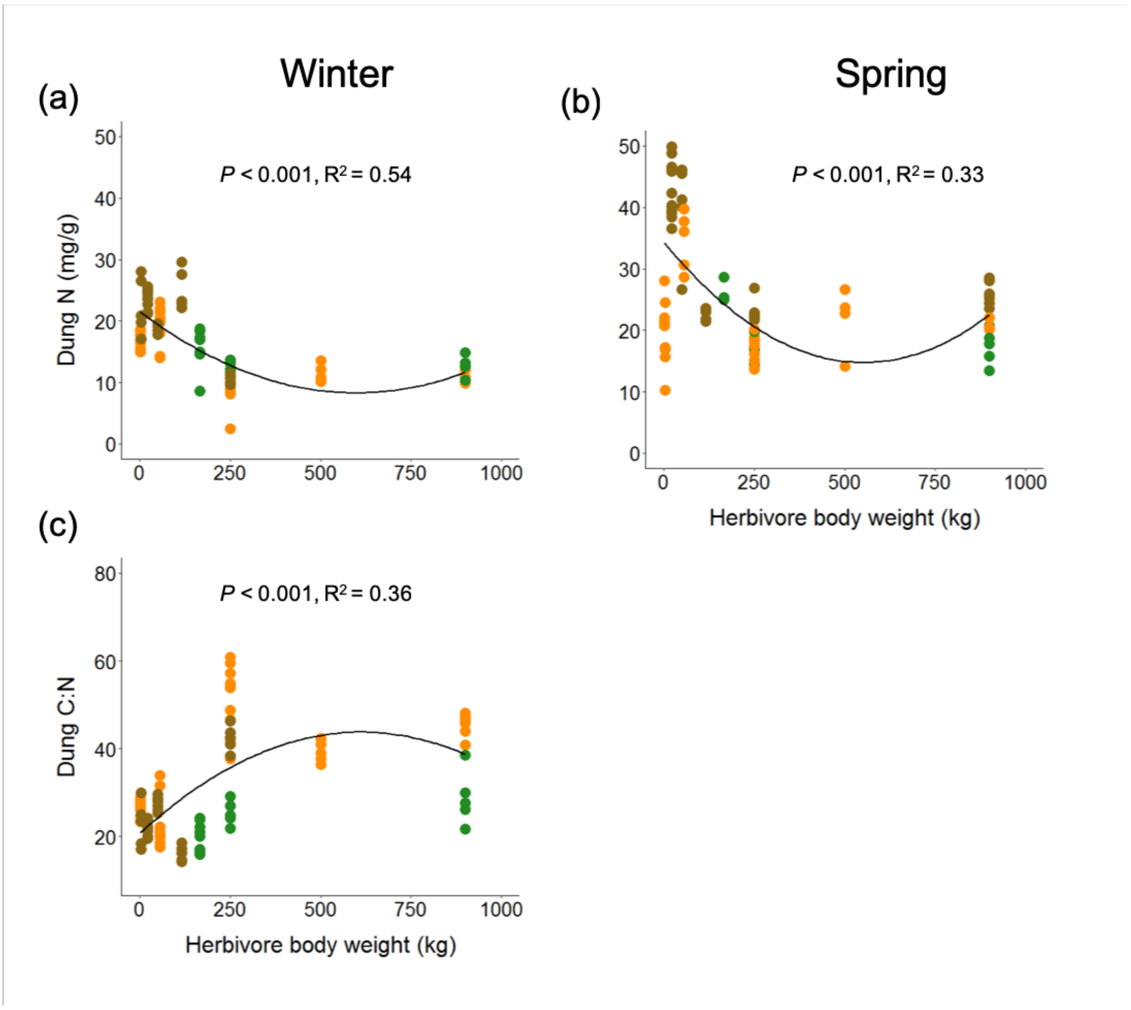


**Supplementary Fig. 1. Dung quality in relation to herbivore body weight.** Relationships between body weight of herbivore species[1] and N concentrations (a, b), and C:N ratios (c) of their dung (mean ± SE; for n per herbivore species see Suppl. Table 1) (orange symbols: Kennemerduinen; brown symbols: Zwarte Beek Vallei; green symbols: Oostvaardersplassen; line: polynomial regression through all dung samples) in winter (a, c) and spring (b). Significant polynomial regression lines are shown.

[1] Smith, FA. *et al*. Body mass of late Quaternary mammals. *Ecology* **84**, 3403–3403 (2003).

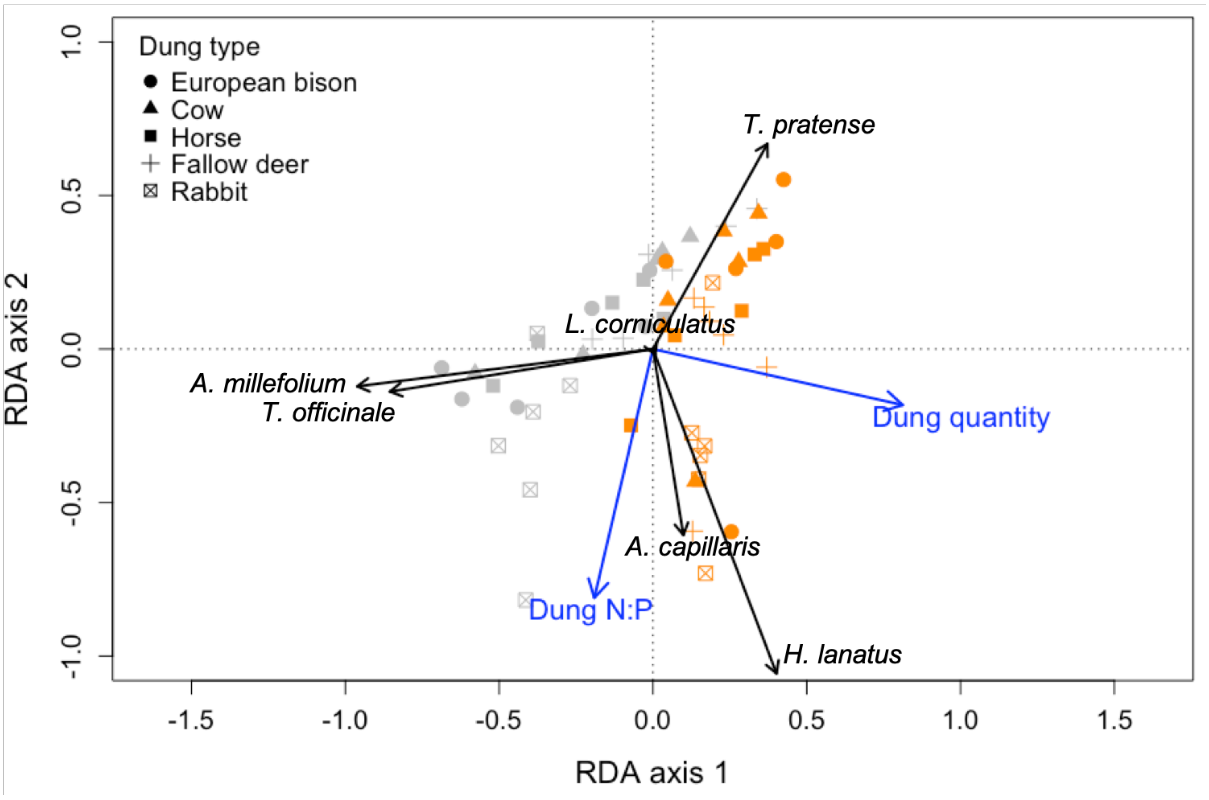


**Supplementary Fig. 2. Ordination plot showing the result of RDA analysis of the relative abundances of plants in mesocosms under different dung treatments.** The dung types are shown with different symbols (see the legend) and the dung quantities with different colours: grey is low quantity (5 g) and orange is high quantity (30 g). Dung N:P and dung quantity are represented by blue arrows and the different plant species by black arrows. The model explained 16.9% of the variance.

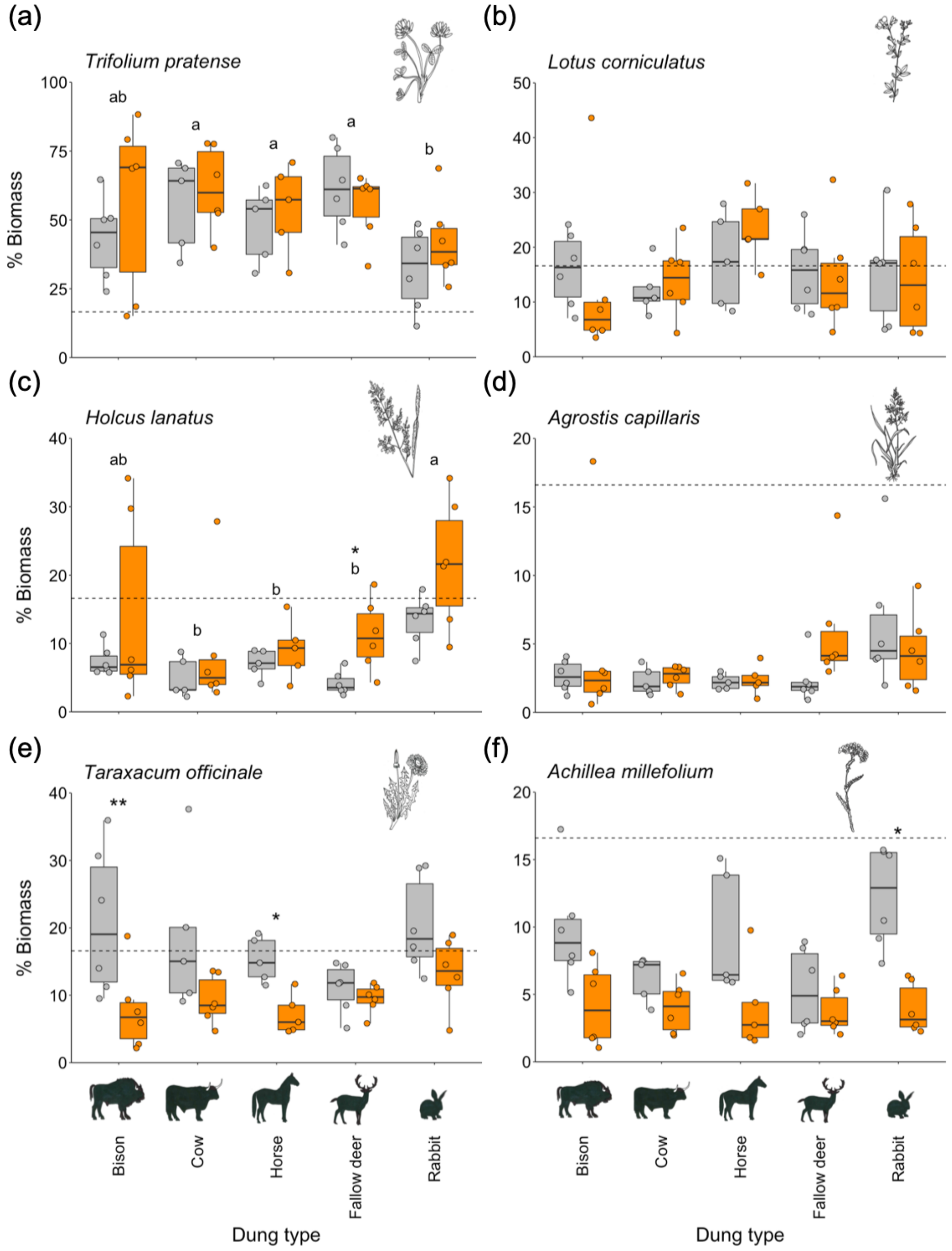


**Supplementary Fig. 3. Effect of dung type and quantity on the relative abundance of the different plant species in an experimental plant community.** Relative abundance of each plant species growing under different dung types and quantities (grey bar: low dung quantity; orange bar: high dung quantity). Bars show means ± SE of 6 replicates, except for low-cow, low-horse and high-horse (n = 5) Values not sharing the same letter indicate significant differences between dung types and asterisks indicate significant differences between dung quantities (results from Tukey-Kramer HSD tests following ANOVA; Suppl. Table 3), with * $P < 0.05$ and ** $P < 0.01$. The dashed lines indicate equal species distribution (16.6 %). Note the different scale of the y-axis in (a).

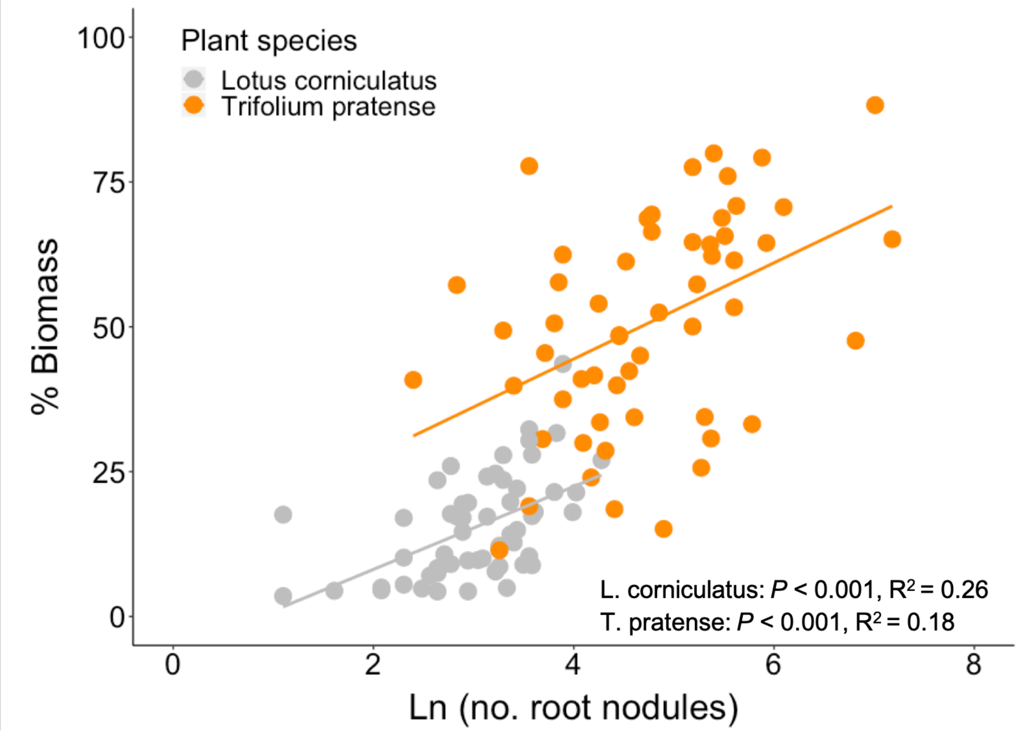


**Supplementary Fig. 4. Legume abundance in relation to nodulation.** Relative abundances of *Lotus corniculatus* (grey points and line) and *Trifolium pratense* (orange points and line) in the artificial plant community plotted against the log-transformed number of root nodules. Significant linear regression lines were drawn for both plant species.

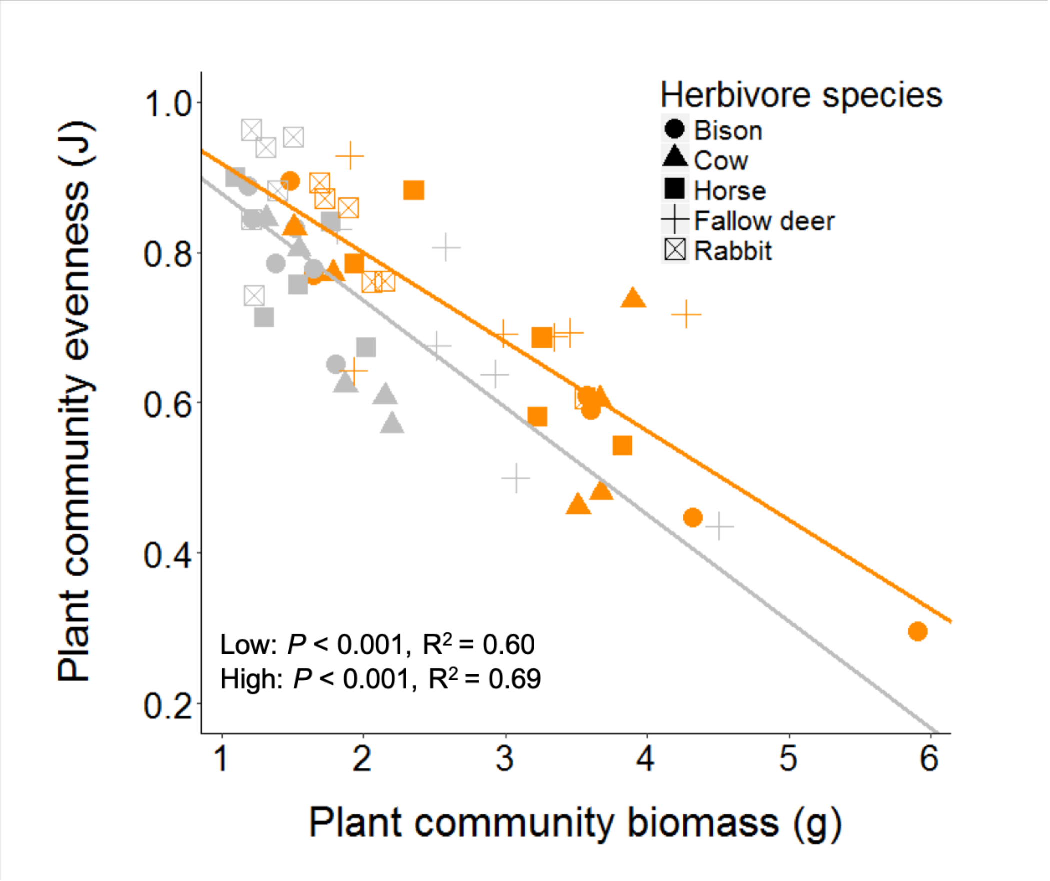


**Supplementary Fig. 5. Plant community evenness plotted against plant community biomass.** Plant communities were growing in mesocosms receiving dung in a low (5 g dried dung; grey points) or a high quantity (30 g dried dung; orange points) from different herbivore species (see different symbols in legend). All plant communities consisted of the same six species with two leguminous forbs (*Trifolium pratense* and *Lotus corniculatus*), two grasses (*Holcus lanatus* and *Agrostis capillaris*), and two other forbs (*Taraxacum officinale* and *Achillea millefolium*). Significant linear regression lines were drawn for both dung quantities.
